# Characterization of Uncultured Genome Fragment from Soil Metagenomic Library Exposed Rare Mismatch of Internal Tetranucleotide Frequency

**DOI:** 10.3389/fmicb.2016.02081

**Published:** 2016-12-22

**Authors:** Yunpeng Liu, Dongqing Yang, Nan Zhang, Lin Chen, Zhongli Cui, Qirong Shen, Ruifu Zhang

**Affiliations:** ^1^Key Laboratory of Microbial Resources Collection and Preservation, Ministry of Agriculture, Institute of Agricultural Resources and Regional Planning, Chinese Academy of Agricultural SciencesBeijing, China; ^2^Jiangsu Key Lab and Engineering Center for Solid Organic Waste Utilization, National Engineering Research Center for Organic-Based Fertilizers, Nanjing Agricultural UniversityNanjing, China; ^3^College of Life Sciences, Nanjing Agricultural UniversityNanjing, China

**Keywords:** uncultured soil bacterium, bacterial artificial chromosome library, 16S rDNA, genome fragment, tetranucleotide correlation

## Abstract

Exploring the genomic information of a specific uncultured soil bacterium is vital to understand its function in the ecosystem but is still a challenge due to the lack of culture techniques. To examine the genomes of uncultured bacteria, a metagenomic bacterial artificial chromosome library derived from a soil sample was screened for 16S rDNA-containing clones. Five clones (4C6, 5E7, 5G4, 5G12, and 5H7) containing uncultured soil bacteria genome fragment (with low 16S rDNA similarity to isolated bacteria) were selected for sequencing. Clone 5E7 and 5G4 showed only 82 and 83% of 16S rDNA identity to known sequences. Phylogenetic analysis of 16S rDNA indicated that 5E7 and 5G4 were potentially from new class of Chloroflexi. Only one-third of the 5G4 open reading frames have significant hits against HMMER. Internal tetranucleotide frequency analysis indicated that the unknown region of 5G4 was poorly correlated with other parts of the clone, indicating that this section might be obtained through lateral transfer. It was suggested that this region rich for unknown genes is under fast evolution.

## Introduction

Soils are dominated by immensely diverse populations of microorganisms that remain largely unexplored (Torsvik and Øvreås, 2002). It is estimated that more than 99% of the microorganisms present in natural environments are not readily cultivable with known cultivation techniques, and this situation will not change until new culture technologies are developed (Streit and Schmitz, 2004; Urich et al., 2008; Yamada and Sekiguchi, 2009). To overcome the limitations of cultivation techniques, culture-independent strategies, especially novel molecular techniques, have been developed (Liles et al., 2003; Zhou et al., 2010; Lundberg et al., 2012; Lalande et al., 2013).

Fingerprinting techniques, including denaturing gradient gel electrophoresis (DGGE) and temperature gradient gel electrophoresis (TGGE), have been successfully used in many diversity studies and shown to be powerful methods for detecting uncultured microbes in soil (Lalande et al., 2013). However, DGGE/TGGE fails to detect minority populations due to inadequate sensitivity. As DGGE/TGGE is a strategy dependent on polymerase chain reaction (PCR), it only shows information of the amplified sequences and limits its function on analyzing unknown sequences. Gene array is a high-throughput metagenomic tool based on DNA hybridization, which is sensitive enough for the analysis of microbial communities and potential gene functions (Zhou et al., 2010; Tu et al., 2014). However, gene array approaches target functional genes but not the genome. Moreover, the identification of unknown genes is difficult for gene array approach due to the dependence on probe hybridization, which is hard to do with unknown sequences. In addition, establishing the connection between microbial diversity and physiological functions, that is, who is doing what, constitutes a fundamental problem (Maron et al., 2008; Prosser, 2015). Streit and Schmitz (2004) declared that metagenomics might be the key to investigating uncultured microbes (Vavourakis et al., 2016). Although the newly developed single-cell sequencing approach provides a method to obtain insights into uncultured microbes efficiently (Rinke et al., 2013), metagenomic library based method is cost-effective and enables high-throughput identification of organismal communities from small amounts of DNA (Williams et al., 2014). In addition, it is still an efficient way to isolate novel genes from uncultured soil and marine microbes (Zheng et al., 2013; Mai et al., 2014; Peng et al., 2014). Previous studies have obtained information related to uncultured microbes from different environmental samples by sequencing DNA libraries and investigating heterologous expression (Rondon et al., 2000; Liles et al., 2003; Kim et al., 2008; Albertsen et al., 2013).

It has been generally agreed that directly cloning large fragments of the genomic DNA from microbes in natural soil provides a strategy for studying the uncultured microbes (Rondon et al., 2000; Liles et al., 2003; Massana et al., 2008; Li et al., 2012). A bacterial artificial chromosome (BAC) vector with the ability to maintain large DNA fragments stably in *Escherichia coli*, has shown some advantages in metagenomic research (Rondon et al., 2000; Liles et al., 2003; Liu et al., 2011). In a previous study, a metagenomic BAC library derived from microorganisms in red soil was constructed, and the cloning, heterologous expression, and purification of a new endo-β-1,4-glucanase gene, *cel*5G, was achieved (Liu et al., 2010, 2011).

Red soils spread widely in the southern China, cover about 2.04 million km^2^ in tropical and subtropical regions of southern China (Guangming et al., 2003; Wilson et al., 2004). Double cropping system of wheat (*Triticum aestivum* L.) and corn (*Zea mays* L.) is dominant in the upland of this agricultural region (Xu et al., 2003). Due to some unfavorable properties, such as low pH and deficiencies of phosphorus, calcium, and magnesium, the productivity of these soils is generally low. In the current study, based on restriction fragment length polymorphism (RFLP) and 16S rDNA sequencing, we isolated five clones with inserts from uncultured bacteria from the red soil-derived metagenomic BAC library. Sequencing of the BAC inserts provided a glimpse of the genomes of these five uncultured bacteria together with the 16S rDNA and showed a rare mismatch of internal tetranucleotide frequency in a clone.

## Materials and Methods

### Metagenomic BAC Library

The metagenomic library containing 3,024 BAC clones was constructed in a previous study (Liu et al., 2010, 2011). The DNA sample was from red soil collected at the Yingtan Red Soil Ecological Station (28°15′20′′ N, 116°55′30′′ E) of the Chinese Academy of Science, Jiangxi Province, China. The BAC library was estimated to contain approximately 200 Mb, with an average insert size of 75 kb. The library was stored at –80°C in 32 96-well cell culture plates containing 200 μl of Luria-Bertani (LB) medium with 12.5 μg/ml chloramphenicol (Cm) and 30% glycerol in each well.

### Plasmid Isolation from the BAC Library

All clones were inoculated into new 96-well plates for activation and then the contents of each well were transferred to 3 ml fresh liquid LB medium with 12.5 μg/ml Cm and cultured overnight. The plasmids were extracted following the protocol described by Liu et al. (2011), and the residual chromosomal DNA from the plasmid host (*E. coli* DH10B) was digested by plasmid-safe, ATP-dependent DNase (Epicentre Technologies) at 37°C for 2 h to remove the nicked DNA. The reactions were then incubated in a water bath at 70°C for 15 min to inactivate the DNase.

### Screening of 16S rRNA Gene-Containing BAC Plasmids

To screen the 16S rRNA gene-containing BAC plasmids in the library, the extracted plasmids and the bacterial cells from the library were used to amplify the 16S rDNA fragment in 96-well PCR plates in a 25 μl volume containing 1 μl of DNA or cell suspension as the template, 2.5 μl of 10 × PCR buffer, 2 μl of Mg^2+^ (20 mM), 2 μl of 2.5 mM dNTP, 1 μl (10 pmol/μl) of each of the primers (27F, 5′-AGAGTTTGATCCTGGCTCAG and 1492R, 5′-GGTTACCTTGTTACGACTT), 0.5 μl of Taq polymerase, and 16 μl of ddH_2_O. The primer pair amplified about 1,500 bp of the 16S rDNA. The reaction program included 5 min of denaturation at 95°C, 30 cycles of 95°C for 1 min, 54°C for 90 s, and extension at 72°C for 120 s followed by 10 min of extension at 72°C. The PCR products amplified from the extracted BAC plasmids were detected on 1% agarose gels.

All the PCR reactions using bacterial cells as templates resulted the amplification of 16S rDNA products of the BAC host, *E. coli* DH10B. To eliminate this background and screen for the 16S rRNA genes contained in the BAC plasmids, the RFLP analysis using endonuclease *HhaI*, which recognizes GCGC sites, was performed for all the PCR products. The digestion was carried out at 37°C for 2 h. The restriction fragments were analyzed on 1% agarose gels, and the 16S rDNA of *E. coli* DH10B was used as the control. The agarose gel was stained with ethidium bromide and analyzed with a digital imaging system. The 16S rDNA products that showed RFLP profiles different with that of the *E. coli* DH10B control were selected and verified by amplification from the corresponding BAC plasmids. In total, 500 BAC clones were screened, and seven 16S rRNA gene-containing BAC plasmids were obtained.

### 16S rRNA Sequencing and Phylogenetic Tree Construction

The confirmed 16S rRNA genes contained in the BAC plasmids were re-amplified with high fidelity polymerase using DNase digested and purified BAC plasmids as templates. The PCR products were purified and linked to the pMD19-T vector (TAKARA, Dalian, China) for sequencing, which was performed by GenScript (Nanjing) Co., Ltd. The sequences obtained were searched against the NCBI nr/nt Database using BLAST. In total, five clones, named 4C6, 5E7, 5G4, 5G12, and 5H7, were found to contain 16S rRNA genes with high dissimilarity to cultured bacteria.

A phylogenetic Maximum Likelihood tree of the 16S rDNA sequences was constructed. 16S rDNA sequences of 20 matched type strains from RDP database were downloaded as references. Both uncultured and isolated strain with the 16S rDNA sequences longer than 1,200 bp were included in sequence match. The duplicates were removed. An out group was selected from the RDP Hierarchy Browser. Multiple alignment of the sequences for each BAC clone was conducted by ClustalW. The evolutionary history was inferred by using the Maximum Likelihood method based on the Tamura–Nei model (Tamura and Nei, 1993). Maximum likelihood trees were then constructed using MEGA 6.0 based on 16S rDNA (Tamura et al., 2013). The phylogeny was tested by 100 of bootstrap replications.

### Sequencing and Annotation of the Selected BAC Inserted Fragments

The inserted fragments of the five selected BAC clones were sequenced using a Roche 454 GS FLX system in the Chinese National Human Genome Center (Shanghai, China). Newbler v2.3 was used to assemble the sequences.

For annotation, the protein-coding genes (CDS) were predicted by Glimmer 3 (Delcher et al., 2007), and pseudogenes and anomalous start/stop codons were identified by GenePRIMP (Pati et al., 2010). Then, all of the genes were manually curated with the genome viewer Artemis (Carver et al., 2008). The functional annotation was carried out using the BLASTP with GenBank’s non-redundant protein databases (nr) (parameters: *E*-value = 1*e* – 5, coverage >60%, and identity >50%). Each gene was functionally classified into the cluster of orthologous groups (COGs) categories using an RPS-BLAST search against the COGs database with an *E*-value of 1*e* – 5 (Tatusov et al., 2003). The domain recognition was carried out with an HMMER search (Johnson et al., 2010) against the PFAM database (version 30.0) (Finn et al., 2015) with an *E*-value of 1*e* – 5. The rRNAs were predicated with RNAmmer 1.2 Server (Lagesen et al., 2007). The annotation was compared with reported soil metagenomes in Integrated Microbial Genomes (IMG) system based on the function profile (Markowitz et al., 2014a,b). Profile of the metagenomes across the functions found in the BAC clones was shown. For each study in IMG, one sample was picked to be included in the comparison. In general, 69 soil metagenomes and 172 functions (pfam) were included.

### Tetranucleotide Frequency Analysis

The internal tetranucleotide correlations of BAC inserted sequences were analyzed following the compositional method described by Teeling (Teeling et al., 2004a,b) using a maximal-order Markov model (Schbath et al., 1995). Fragments were extended with their reverse complements. The extended sequences were cut into 300 bp fragments from the beginning of the sequences, with a step size of 100 bp. The frequencies of all 256 tetranucleotides and their corresponding expected frequencies were calculated for these sequences. The frequencies were transformed into *z*-scores for each tetranucleotide. The Pearson correlation coefficients for the *z*-scores were calculated. For each insert, the tetranucleotide frequencies of all the fragments were determined. These results were then directly used for the Pearson correlation analysis. All the above processes were performed by Perl script (The script was shared in Github^1^), and the visual outputs were finished by R script (heatmap.2).

### Accession Numbers

Sequences have been deposited in GenBank with accession number JX091737, JX091738, JX091739, JX091740, and JX091741 corresponding to 16S rDNA sequences of 4C6, 5E7, 5G4, 5G12, and 5H7; KT342854, KT342855, KT342856, KT342857, and KT342858 corresponding to full length sequences of 4C6, 5E7, 5G4, 5G12, and 5H7, respectively. IMG ID for 4C6, 5E7, 5G4, 5G12, and 5H7 are 2695420969, 2695420970, 2695420984, 2695421012, and 2695421011.

## Results

### 16S rDNA Analysis and Sequencing of BAC Inserts

The BAC library was estimated to contain approximately 200 Mb, with an average insert size of 75 kb. As a result, seven clones (4C6, 5E7, 5G4, 5G12, 5H7, 10D9, and 27A5) from 500 clones screened were estimated to include 16S rDNA fragments within the inserts. The 27A5 and 10D9 clones, which showed similarities to known species, were clustered with a *Bacillus* spp. group and a *Pseudomonas* spp. group, respectively. Subsequent sequencing of the 16S rDNA confirmed that five clones, 4C6, 5E7, 5G4, 5G12, and 5H7, were originated from uncultured bacteria. The identities to the nearest BLAST results from cultivable microbes in the NCBI database were 94% (4C6), 82% (5E7), 83% (5G4), 88% (5G12), and 97% (5H7) (**Table 1**). The 5E7 and 5G4 clones had the lowest similarities to all known 16S rDNA sequences and showed interesting differences from the cultivable microbes in the subsequent analysis. Clones containing ambiguous 16S rDNA were removed in the screening step, which led to a much lower proportion of positive clones in the library than expected.

**Table 1:** General information of five BAC inserts.

	4C6	5E7	5G4	5G12	5H7
BAC insert length (bp)	23,678	46,092	30,180	43,604	56,420
G + C content (%)	51.6	49.6	48.2	49.2	49.0
No. of predicted ORF	18	44	36	42	59
No. of hypothetical protein	8	6	19	14	14
Proteins assigned to COGs	7	32	12	29	39
Average ORF length (bp)	1,017	981	617	912	788
Coding regions (%)	54.4	75.6	44.4	58	65
rrn operon	16S	16S-23S-5.8S	16S-23S	16S-23S-5.8S	16S-23S-5.8S
Nearest relation (accession no.)	HQ118747.1	EF516466.1	FJ479355.1	KC555030.1	FJ820395.1
	Uncultured	Uncultured	Uncultured	Uncultured	Uncultured
Identities to cultivable sample^a^	97% (94%)	82% (82%)	83% (83%)	93% (88%)	98% (97%)

^a^*Numbers in brackets indicate 16S rDNA identities to known genomes.*

**FIGURE 1:**
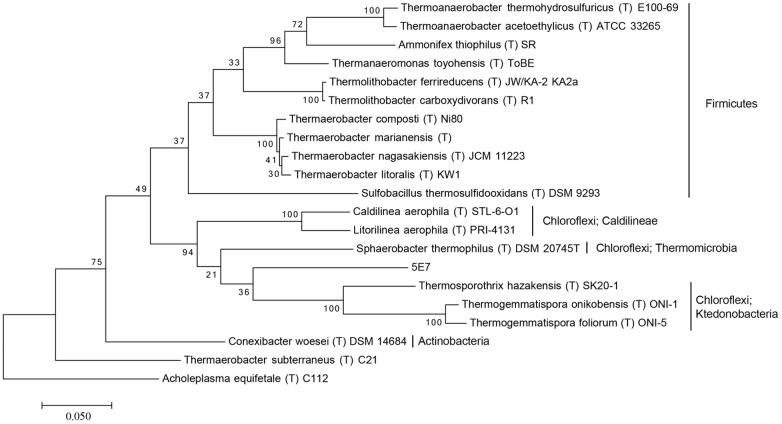
**Molecular phylogenetic analysis of 5E7 by Maximum Likelihood method.** The evolutionary history was inferred by using the Maximum Likelihood method based on the Tamura–Nei model. The tree with the highest log likelihood (-10636.1388) is shown. Initial tree for the heuristic search were obtained automatically by applying Neighbor-Join and BioNJ algorithms to a matrix of pairwise distances estimated using the Maximum Composite Likelihood (MCL) approach, and then selecting the topology with superior log likelihood value. The tree is drawn to scale, with branch lengths measured in the number of substitutions per site. The analysis involved 21 nucleotide sequences. All positions containing gaps and missing data were eliminated. There were a total of 1,301 positions in the final dataset. Evolutionary analyses were conducted in MEGA7. The phylogeny was tested by 100 of bootstrap replications.

**FIGURE 2:**
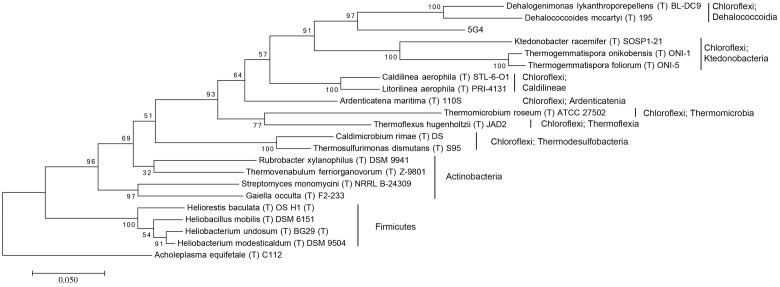
**Molecular phylogenetic analysis of 5G4 by Maximum Likelihood method.** The evolutionary history was inferred by using the Maximum Likelihood method based on the Tamura–Nei model. The tree with the highest log likelihood (-11781.7674) is shown. Initial tree for the heuristic search were obtained automatically by applying Neighbor-Join and BioNJ algorithms to a matrix of pairwise distances estimated using the MCL approach, and then selecting the topology with superior log likelihood value. The tree is drawn to scale, with branch lengths measured in the number of substitutions per site. The analysis involved 22 nucleotide sequences. All positions containing gaps and missing data were eliminated. There were a total of 1,244 positions in the final dataset. Evolutionary analyses were conducted in MEGA7. The phylogeny was tested by 100 of bootstrap replications.

For the investigation of the phylogenetic position of the five uncultured BAC clones within the bacterial domain, a phylogenetic tree based on 16S rRNA gene sequences was constructed (**Figures 1** and **2**, Supplementary Figures S1–S3). The phylogenetic tree of the five uncultured clones showed that 4C6 belonged to Mucilaginibacter (Figure S1), 5H7 belonged to the Novosphingobium (Figure S2) and 5G12 was clustered with the Gaiella (Figure S3). 5E7 and 5G4 were clustered with the Chloroflexi, however, both of them showed difference with known classes (**Figures 1** and **2**). Note that the bootstrap values above 5E7 were low, the reason is that the similarity between 5E7, *Sphaerobacter thermophilus* (T) DSM20745T and the *Ktedonobacteria* is similar. Also the analysis using RDP classifier showed 5E7 and 5G4 were from unknown classes of Chloroflexi [5E7: Bacteria (100%) “Chloroflexi” (96%) Caldilineae (30%) Caldilineales (30%) Caldilineaceae (30%) Litorilinea (30%); 5G4: Root (100%) Bacteria (100%) “Chloroflexi” (99%) Dehalococcoidia (88%) Dehalococcoidales (88%) Dehalococcoidaceae (88%) Dehalococcoides (88%)] (Wang et al., 2007). The highest identities of 5E7 and 5G4 to known species were only 82% and 83%, respectively (**Table 1**). To our knowledge, this is the first report of genomic fractions of bacteria with such low identities to known species. A similar result, i.e., that cultured and uncultured bacteria differed greatly in a phylogenetic analysis of 16S rRNA genes, has been reported previously (Suzuki et al., 1997; Cottrell et al., 2000).

**FIGURE 3:**
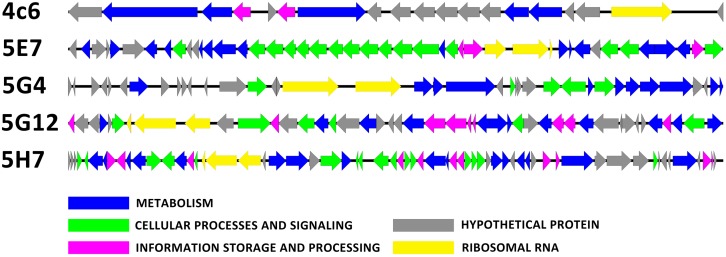
**The open reading frame map.** The annotated ORFs are drawn with different colors of arrows based on the COG classification.

The inserted fragments of the five uncultured BAC clones (4C6, 5E7, 5G4, 5G12, and 5H7) were completely sequenced. The length of the inserts ranged from 23.7 to 56.4 kbp. The percentages of G + C were 51.6, 49.6, 48.2, 49.2, and 49.0 for 4C6, 5E7, 5G4, 5G12, and 5H7, respectively. For the organization of the rRNA, 5E7, 5G12, and 5H7 all contained a 5.8S-23S-16S operon, whereas 5G4 had a 23S-16S operon, and 4C6 had a single 16S rRNA gene. Annotation of the inserted fragments predicted 18 open reading frames (ORFs) for 4C6, 44 for 5E7, 36 for 5G4, 42 for 5G12, and 59 for 5H7. The annotations have been uploaded to IMG system with the ID of 2695420969, 2695420970, 2695420984, 2695421012, and 2695421011 for 4C6, 5E7, 5G4, 5G12, and 5H7. The functions (pfam) found in these clones were compared with the reported soil metagenomes to show the frequency of these functions in other soil metagenomes (**Supplementary Table S2**). The number of predicted ORFs assigned to the COGs categories was seven for 4C6, 32 for 5E7, 12 for 5G4, 29 for 5G12, and 39 for 5H7 (**Figure 3**; **Table 1**). The 5E7 clone was rich in genes related to cellular processes and signaling (**Figure 3**). The distribution of genes in 5G12 and 5H7 was uniform, but 4C6 was too short to give an overview of the trend (**Figure 3**). The hypothetical proteins ranged from 6 to 19 (**Table 1** and Supplementary Table S1). Interestingly, 5G4 contained 19 hypothetical proteins in 36 predicted ORFs, and only one-third of the ORFs were annotated with known functions (**Table 1** and Supplementary Table S1). The proportions of unknown ORFs that could not be assigned to COGs, were 61.1%, 21.9%, 66.6%, 30.9%, and 33.8% for 4C6, 5E7, 5G4, 5G12 and 5H7, respectively. Interestingly, 13 unknown genes in 5G4 were assembled together (located between 3 nt and 9,792 nt of the insert). Because 5G4 is from an uncultured bacterium with a phylogenetic relationship distant from all known bacteria (83%), it will be interesting and important to investigate further the functions of these unknown genes. In contrast to 5G4, another clone, 5E7, with 83% identity to known species, enjoyed a clear gene annotation and only 21.9% of the genes could not be assigned to COGs.

### Tetranucleotide Frequency Correlations

To obtain a deeper understanding of the genome, an internal tetranucleotide preference analysis was performed for the five clones. It has been reported that the tetranucleotide frequencies of genomic DNA sequences are highly conserved (Noble et al., 1998). Tetranucleotide preference is a widely used genome signature to identify genomes (Teeling et al., 2004a,b), and it has already been used for metagenomic analysis of fosmid inserts (Li et al., 2012).

**FIGURE 4:**
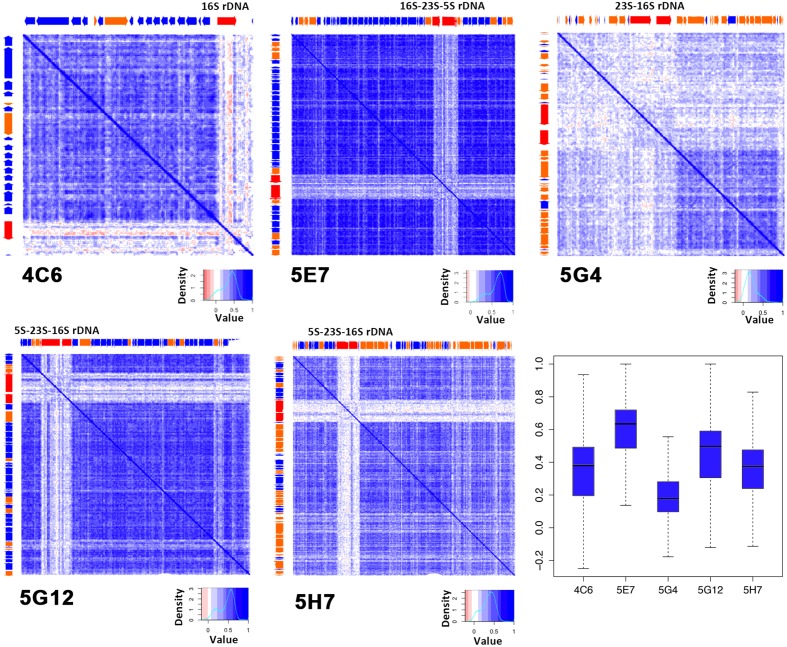
**Internal tetranucleotide frequency correlations of five colors indicate the tetranucleotide correlation between each of the 300 bp fragments.** Deep blue represents high correlation regions. ORF positions are shown on the top and left sides of each map. The boxplot shows the summary of the correlation matrix.

Exogenous sequences contained in the inserted fragments should show low correlations with the local part and could be detected by a heatmap (Supplementary Figure S4). Here, we performed the internal tetranucleotide correlations analysis with a newly developed program written with Perl and based on the algorithm reported previously (Teeling et al., 2004a). Generally, a lighter map indicates an unstable genome with many exogenous sequences. The *R*-values are summarized in a boxplot (**Figure 4**). The internal tetranucleotide frequency correlation map of 5E7 exhibited overwhelmingly high stabilities with an *R*-value of 0.59 ± 0.18; 5G12 also revealed a high internal correlation of tetranucleotide frequency and a high coding region percentage. In contrast, 5G4 was highly unstable with an average *R*-value of 0.20 ± 0.16 (less than 0.6). The region rich for unknown genes was highly unstable (**Figure 4**; Supplementary Table S1). It should be noted that the sections of rRNA were visibly different from the rest of genome as reported before (Noble et al., 1998). We suggest that the host of 5G4 was frequently transformed with motile DNA from other organisms, and the functions of these genes were not known. This hypothesis supports the low coding percentage of 5G4 (**Table 1**). Due to the insertion of exogenous DNA, endogenous genes were inactivated, and as a result, the left region became a non-coding region. In contrast, 5E7 contained a potentially stable genome with less exogenous DNA and a high proportion of coding region. In addition, the coding region was highly correlated with the R-value of the internal tetranucleotide correlation for all these five clones (*R* = 0.895, *p* = 0.039).

## Discussion

In current study, by using an RFLP method, the metagenomic library containing 3,024 BAC clones from the DNA sample from red soil in South China constructed in a previous study (Liu et al., 2011) was screened for uncultured bacterial insertions. Finally, two uncultured clones 5G4 and 5E7 were isolated with low identities of 16S rDNA (83 and 82%) to known bacteria. The phylogenetic analysis showed that both of them belong to a new class in Chloroflexi. However, while 5E7 enjoys a clear and highly self-correlated genome fragment as supposed, 5G4 is rich for unknown genes and has an unstable genome, which suggest the frequent lateral gene transfer in this bacterium.

The findings demonstrated our limited knowledge of soil microbes, especially of functional genes in uncultured bacteria (Prosser, 2015). Some of the uncultured microbial sequences, including 4C6, 5E7, 5G12, and 5H7, are similar to sequences of known species; nevertheless, some of them, such as 5G4, are still beyond our understanding, and many unknown genes are waiting to be identified and classified. Interesting is, 5G4 has extremely low coding region and rich for unknown genes, and 5G4 has a low internal tetranucleotide correlation which indicate an instable genome. Based on these results, we suggest 5G4 with a lot of exogenous genes. Because the insertion of exogenous DNA into the genome would cause a disruption of local genes, which make the rate of coding region lower (**Table 1**). It is suggested that the genome of host bacteria of 5G4 is in fast evolution because acquisition of laterally transferred DNA is much more efficiency than nucleotide substitution in nature, and the former is the primary driver of bacterial speciation (Ochman and Bergthorsson, 1995; Bao et al., 2014). That would explain the low identity of 16S rDNA sequence of 5G4 to that of known bacteria (**Table 1**; **Figure 2**). It is interesting to explore the difference in function even for the other unknown genes in 5G4.

The strategy developed in this research could be applied to the identification and study of uncultured bacterial genes. Although the genomes of a few uncultured microbes have already been completed, or nearly completed by single cell sequencing or metagenomic sequencing (Albertsen et al., 2013), searching for unknown genes from genomes of uncultured microbes was difficult due to the lack of targeted selection of the strains from the microbial mixtures. This strategy provided a deeper view of uncultured bacterial genomes. The 23–56 kbp fragments gave a substantial amount of information about the uncultured bacteria, and the fragments were large enough for tetranucleotide analysis to identify the signatures of the genomes. This information provides pre-isolation of the interested genomes, which might be further targets for sequencing. The single cell sequencing approach is efficient in getting large draft genome of uncultured microbes (de Jager and Siezen, 2011; Rinke et al., 2013), however, the selection of the microbes to be sequenced is generally based on identification of the marker genes, such as 16S rDNA for bacteria (de Jager and Siezen, 2011). In current study, we showed a better way to find interesting or rare genomes from uncultured microbes, which could provide better perspective to select genomes to seq, i.e., that selecting a genome with one of the unknown genes in clone 5G4.

In general, these sequences give initial information to understand the host bacteria, and possibility to hybridize the larger genome fragment of these uncultured bacteria with interest in soil purposely.

## Author Contributions

ZC, QS, and RZ designed the experiment. YL and LC performed the screening of the uncultured clones and the tetranucleotide analysis. DY and NZ performed the genomic analyses and annotations.

## Conflict of Interest Statement

The authors declare that the research was conducted in the absence of any commercial or financial relationships that could be construed as a potential conflict of interest.

## Footnotes

1 https://github.com/YunpengLiu/Tetra-nucleotide-analysis

## Supplementary Material

TABLE S2 **Frequency of each ORF in metagenomics in IMG**.
