# Supplementary material for: Characterization of Uncultured Genome Fragment from Soil Metagenomic Library Exposed Rare Mismatch of Internal Tetranucleotide Frequency
Source: Front Microbiol. 2016 Dec 22;7:2081. doi: 10.3389/fmicb.2016.02081 (PMC5177636; doi:10.3389/fmicb.2016.02081)
Supplement: Supplementary file 1 [file Supplementary_Materials.DOC]

**Supplemental materials**

**Phylogenetic analysis**

Phylogenetic maximum likelihood trees of the 16S rDNA sequences was constructed. 16S rDNA sequences of 20 matched type strains from RDP database were downloaded as references. Both uncultured and isolated strain with the 16S rDNA sequences longer than 1200bp were included in sequence match. The duplicates were removed. An out group was selected from the RDP Hierarchy Browser. Multiple alignment of the sequences for each BAC clone was conducted by ClustalW. The evolutionary history was inferred by using the Maximum Likelihood method based on the Tamura-Nei model. Maximum likelihood trees were then constructed using MEGA 6.0 based on 16S rDNA (Tamura et al. 2013). The phylogeny was tested by 100 of bootstrap replications.


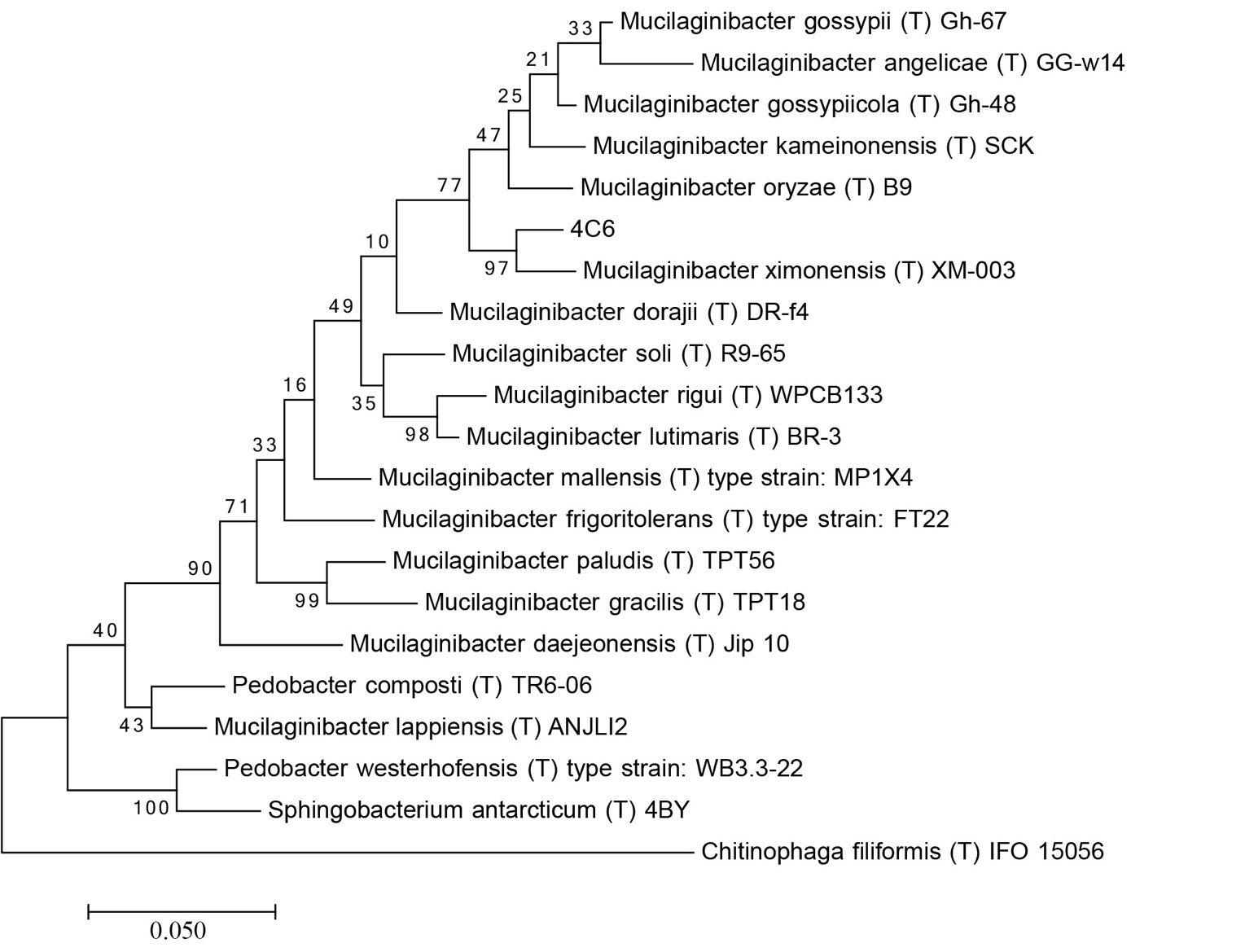


Figure S1. Molecular Phylogenetic analysis of 4C6 by Maximum Likelihood method

The evolutionary history was inferred by using the Maximum Likelihood method based on the Tamura-Nei model. The tree with the highest log likelihood (-6858.1269) is shown. Initial tree(s) for the heuristic search were obtained automatically by applying Neighbor-Join and BioNJ algorithms to a matrix of pairwise distances estimated using the Maximum Composite Likelihood (MCL) approach, and then selecting the topology with superior log likelihood value. The tree is drawn to scale, with branch lengths measured in the number of substitutions per site. The analysis involved 21 nucleotide sequences. All positions containing gaps and missing data were eliminated. There were a total of 1311 positions in the final dataset. Evolutionary analyses were conducted in MEGA7. The phylogeny was tested by 100 of bootstrap replications.


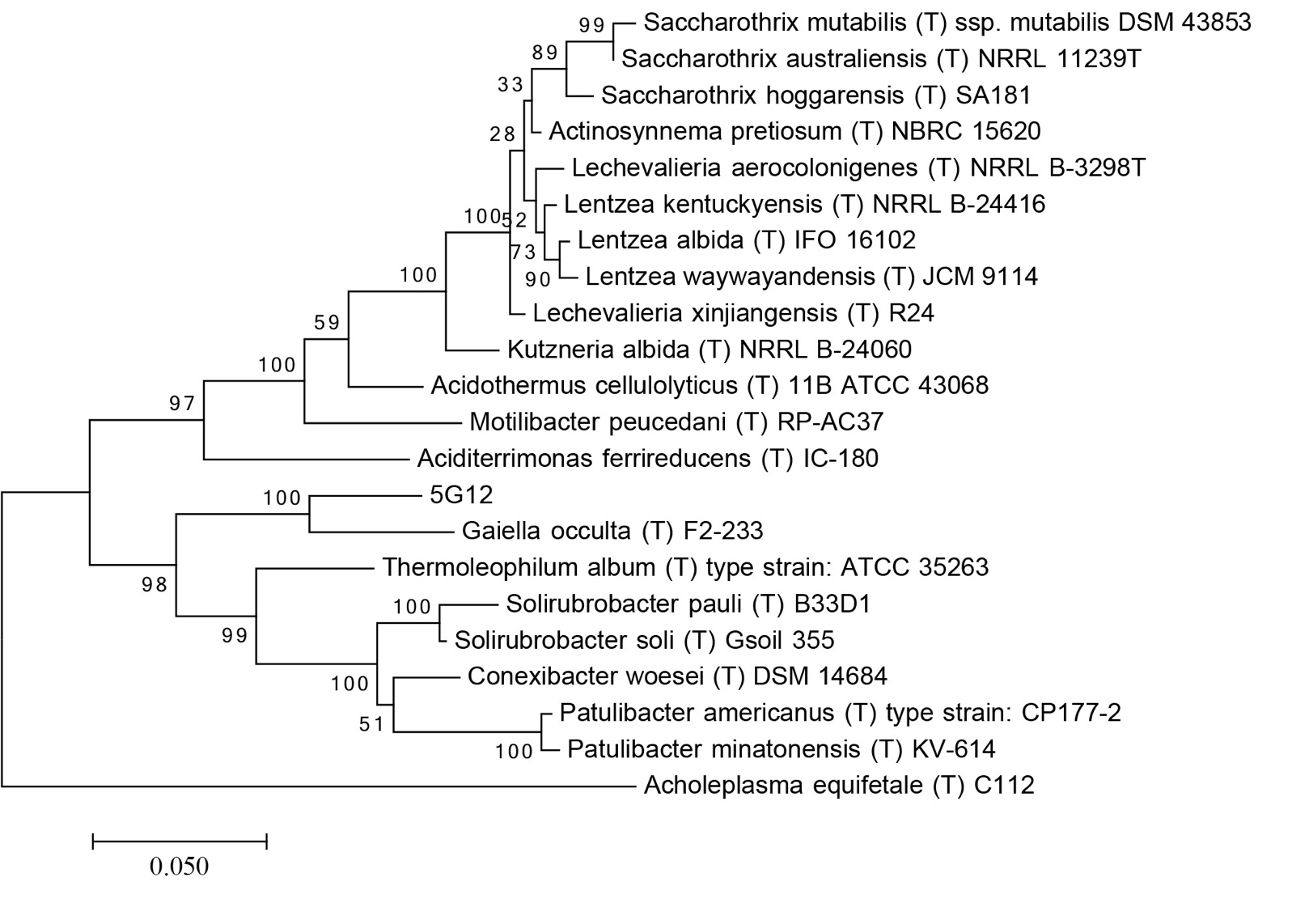


Figure S2. Molecular Phylogenetic analysis of 5G12 by Maximum Likelihood method

The evolutionary history was inferred by using the Maximum Likelihood method based on the Tamura-Nei model. The tree with the highest log likelihood (-7130.3522) is shown. Initial tree(s) for the heuristic search were obtained automatically by applying Neighbor-Join and BioNJ algorithms to a matrix of pairwise distances estimated using the Maximum Composite Likelihood (MCL) approach, and then selecting the topology with superior log likelihood value. The tree is drawn to scale, with branch lengths measured in the number of substitutions per site. The analysis involved 22 nucleotide sequences. All positions containing gaps and missing data were eliminated. There were a total of 1269 positions in the final dataset. Evolutionary analyses were conducted in MEGA7. The phylogeny was tested by 100 of bootstrap replications.


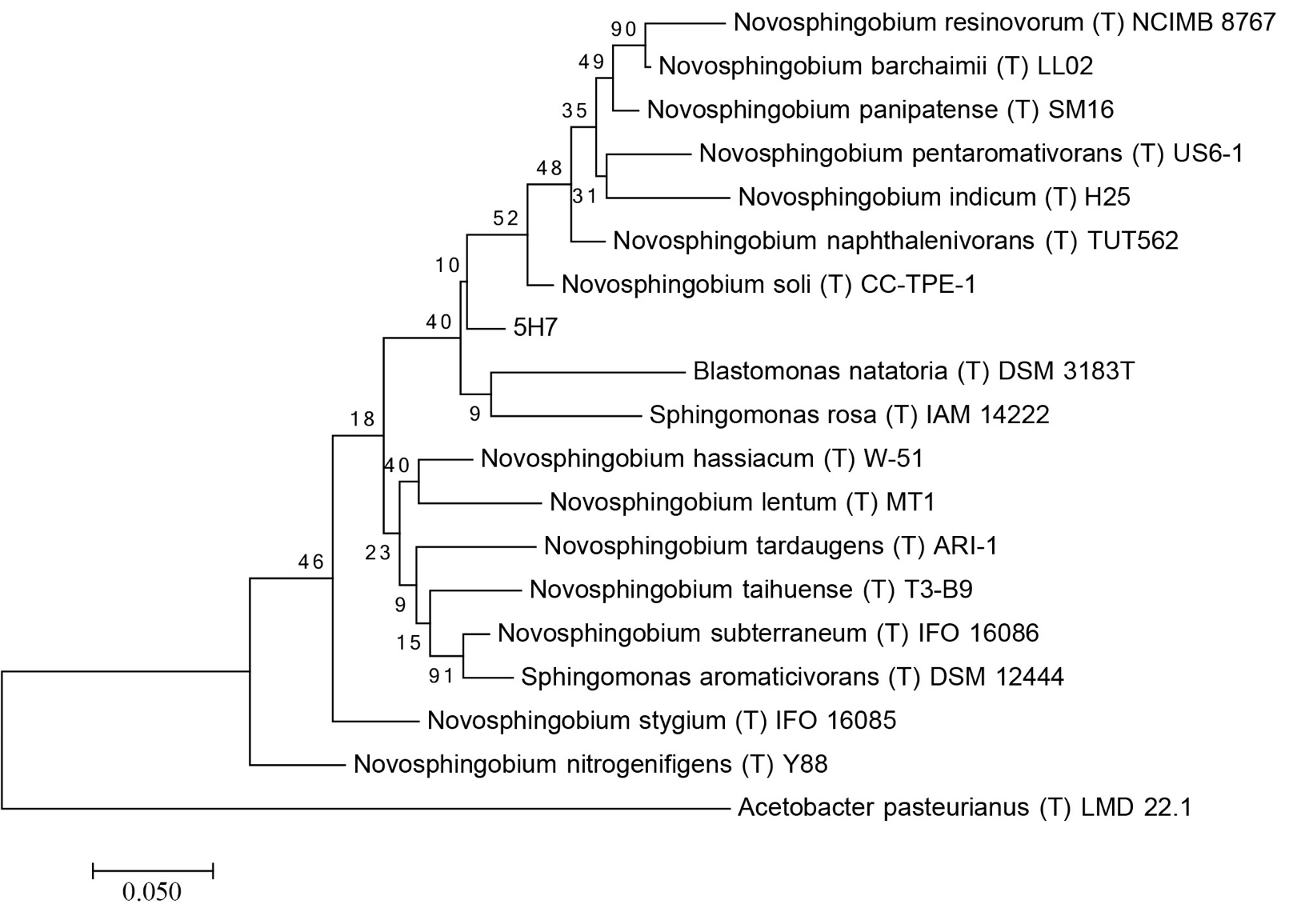


Figure S3 Molecular Phylogenetic analysis of 5H6 by Maximum Likelihood method

The evolutionary history was inferred by using the Maximum Likelihood method based on the Tamura-Nei model. The tree with the highest log likelihood (-5087.8586) is shown. Initial tree(s) for the heuristic search were obtained automatically by applying Neighbor-Join and BioNJ algorithms to a matrix of pairwise distances estimated using the Maximum Composite Likelihood (MCL) approach, and then selecting the topology with superior log likelihood value. The tree is drawn to scale, with branch lengths measured in the number of substitutions per site. The analysis involved 19 nucleotide sequences. All positions containing gaps and missing data were eliminated. There were a total of 1293 positions in the final dataset. Evolutionary analyses were conducted in MEGA7. The phylogeny was tested by 100 of bootstrap replications.

**Tetranucleotide frequency analysis**

For tetranucleotide frequency analysis, the BAC inserted sequences were searched against the NCBI genome database using BLAST, and the top 5 matched genomes were downloaded and used as references in the tetranucleotide frequency analysis following the compositional method described by Teeling (Teeling et al. 2004a; Teeling et al. 2004b) using a maximal-order Markov model (Schbath et al. 1995). Fragments and genomes were extended with their reverse complements. The frequencies of all 256 tetranucleotides and their corresponding expected frequencies were calculated for these sequences. For the correlation between different fragments, the frequencies were transformed into z-scores for each tetranucleotide. The Pearson correlation coefficients for the z-scores were calculated.


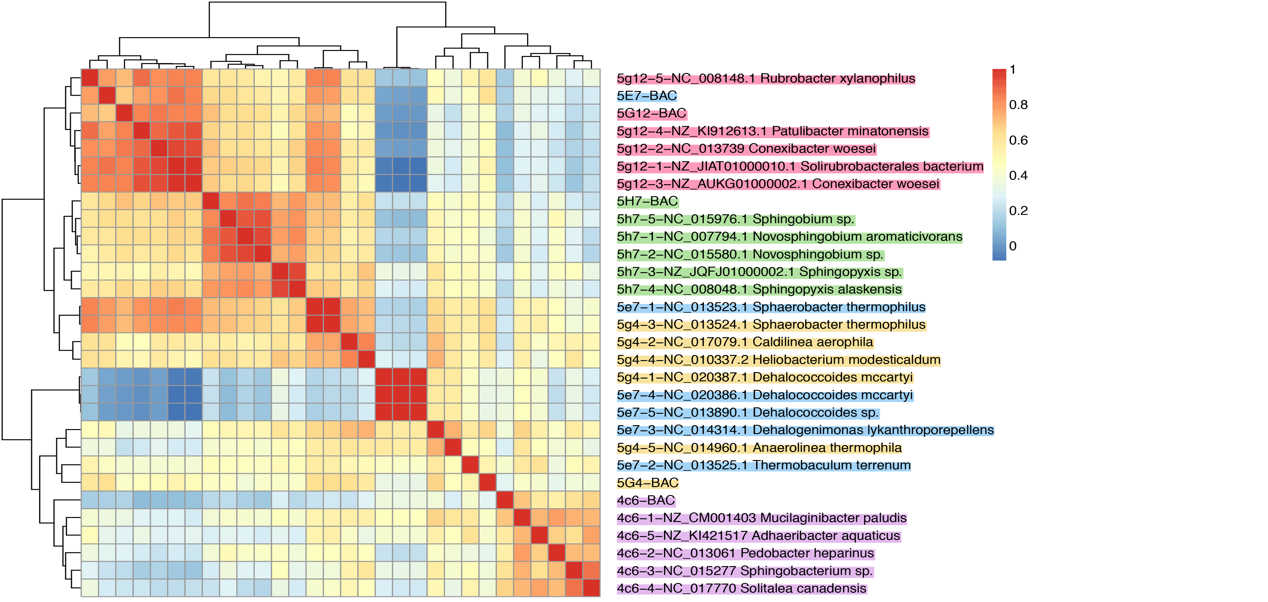


Figure S4 Tetranucleotide correlations of five uncultured BAC inserts. red blocks indicate high correlations between the two genomes, blue blocks indicate low correlations. Prefix of the genome name such as 4C6 indicate that its 16S rDNA sequence has a high identity to that of 4C6. This map indicates that the tetranucleotide preference between genome with similar 16S rDNA sequence is highly correlated.

**Annotations**

Table S1 The detailed annotation information for the five uncultured inserts.

| PID | start | stop | Hmm acc and name^a^ |
| --- | --- | --- | --- |
| 4C6_001 | 1209 | 1 | PF14322.4,SusD-like_3 |
| 4C6_002 | 4672 | 1220 | PF13715.4,CarbopepD_reg_2;PF07715.13,Plug |
| 4C6_003 | 5915 | 4818 | PF04773.11,FecR;PF16344.3,DUF4974 |
| 4C6_004 | 6594 | 5944 | PF04542.12,Sigma70_r2;PF08281.10,Sigma70_r4_2 |
| 4C6_005 | 7231 | 7548 | hypothetical protein |
| 4C6_006 | 8198 | 7545 | PF01325.17,Fe_dep_repress;PF02742.13,Fe_dep_repr_C;PF04023.12,FeoA; |
| 4C6_007 | 8299 | 10767 | PF13715.4,CarbopepD_reg_2;PF07715.13,Plug;PF00593.22,TonB_dep_Rec; |
| 4C6_008 | 11313 | 10801 | hypothetical protein |
| 4C6_009 | 12375 | 11635 | hypothetical protein |
| 4C6_010 | 13249 | 12479 | hypothetical protein |
| 4C6_011 | 14065 | 13256 | hypothetical protein |
| 4C6_012 | 14873 | 14073 | hypothetical protein |
| 4C6_013 | 15629 | 14880 | hypothetical protein |
| 4C6_014 | 16646 | 15747 | PF16119.3,DUF4835 |
| 4C6_015 | 17848 | 16646 | PF02441.17,Flavoprotein;PF04127.13,DFP |
| 4C6_016 | 18277 | 17939 | PF01192.20,RNA_pol_Rpb6 |
| 4C6_017 | 19219 | 18317 | PF13525.4,YfiO |
| 4C6_019 | 19960 | 21470 | rRNA |
| 4C6_019 | 23676 | 23446 | hypothetical protein |
| 5E7_001 | 576 | 73 | hypothetical protein |
| 5E7_002 | 1537 | 899 | PF02230.14,Abhydrolase_2 |
| 5E7_003 | 1680 | 2699 | PF12740.5,Chlorophyllase2 |
| 5E7_004 | 2709 | 2942 | hypothetical protein |
| 5E7_005 | 2954 | 3685 | PF00881.22,Nitroreductase |
| 5E7_006 | 3851 | 5392 | hypothetical protein |
| 5E7_007 | 6255 | 5389 | PF00127.18,Copper-bind |
| 5E7_008 | 7242 | 6766 | PF08239.9,SH3_3 |
| 5E7_009 | 8276 | 7320 | PF00144.22,Beta-lactamase |
| 5E7_010 | 8683 | 8318 | PF00903.23,Glyoxalase |
| 5E7_011 | 9193 | 8693 | hypothetical protein |
| 5E7_012 | 9453 | 9205 | PF02325.15,YGGT |
| 5E7_013 | 10152 | 9463 | PF01168.18,Ala_racemase_N |
| 5E7_014 | 11794 | 10163 | PF04055.19,Radical_SAM |
| 5E7_015 | 12625 | 11861 | PF08282.10,Hydrolase_3 |
| 5E7_016 | 13854 | 12706 | PF00091.23,Tubulin;PF12327.6,FtsZ_C |
| 5E7_017 | 15119 | 13881 | PF14450.4,FtsA;PF02491.18,SHS2_FTSA;PF14450.4,FtsA |
| 5E7_018 | 16017 | 15169 | PF08478.8,POTRA_1 |
| 5E7_019 | 16932 | 16018 | PF01565.21,FAD_binding_4;PF02873.14,MurB_C |
| 5E7_020 | 18197 | 16929 | PF01225.23,Mur_ligase;PF08245.10,Mur_ligase_M;PF02875.19,Mur_ligase_C |
| 5E7_021 | 19326 | 18298 | PF03033.18,Glyco_transf_28;PF04101.14,Glyco_tran_28_C |
| 5E7_022 | 20485 | 19268 | PF01098.17,FTSW_RODA_SPOVE |
| 5E7_023 | 21810 | 20482 | PF08245.10,Mur_ligase_M |
| 5E7_024 | 22760 | 21807 | PF10555.7,MraY_sig1;PF00953.19,Glycos_transf_4 |
| 5E7_025 | 24201 | 22828 | PF01225.23,Mur_ligase;PF08245.10,Mur_ligase_M;PF02875.19,Mur_ligase_C |
| 5E7_026 | 26090 | 24201 | PF03717.13,PBP_dimer;PF00905.20,Transpeptidase |
| 5E7_027 | 26511 | 26083 | PF04999.11,FtsL |
| 5E7_028 | 27433 | 26516 | PF01795.17,Methyltransf_5 |
| 5E7_029 | 27810 | 27436 | PF02381.16,MraZ;PF02381.16,MraZ |
| 5E7_030 | 27851 | 29197 | PF00675.18,Peptidase_M16;PF05193.19,Peptidase_M16_C |
| 5E7_031 | 29353 | 30833 | rRNA |
| 5E7_032 | 31168 | 34237 | rRNA |
| 5E7_033 | 34355 | 34471 | rRNA |
| 5E7_034 | 34528 | 35238 | PF13561.4,adh_short_C2 |
| 5E7_035 | 35270 | 35455 | hypothetical protein |
| 5E7_036 | 36735 | 35572 | PF02515.15,CoA_transf_3; |
| 5E7_037 | 37451 | 36738 | hypothetical protein |
| 5E7_038 | 38689 | 37466 | PF03972.12,MmgE_PrpD |
| 5E7_039 | 40155 | 38800 | PF09587.8,PGA_cap |
| 5E7_040 | 41276 | 40161 | PF09084.9,NMT1 |
| 5E7_041 | 41288 | 42793 | PF00501.26,AMP-binding;PF13193.4,AMP-binding_C |
| 5E7_042 | 43760 | 42798 | PF00296.18,Bac_luciferase |
| 5E7_043 | 43899 | 44747 | PF01261.22,AP_endonuc_2 |
| 5E7_044 | 44819 | 46090 | PF08448.8,PAS_4;PF00990.19,GGDEF |
| 5G4_001 | 3 | 173 | hypothetical protein |
| 5G4_002 | 320 | 225 | hypothetical protein |
| 5G4_003 | 1084 | 1473 | hypothetical protein |
| 5G4_004 | 1793 | 1518 | hypothetical protein |
| 5G4_005 | 1864 | 2007 | hypothetical protein |
| 5G4_006 | 2718 | 2371 | hypothetical protein |
| 5G4_007 | 2833 | 3696 | PF02900.16,LigB |
| 5G4_008 | 4290 | 4682 | PF06197.11,DUF998 |
| 5G4_009 | 5031 | 5219 | hypothetical protein |
| 5G4_010 | 5245 | 5490 | hypothetical protein |
| 5G4_011 | 5676 | 5500 | hypothetical protein |
| 5G4_012 | 6405 | 6271 | hypothetical protein |
| 5G4_013 | 6981 | 8228 | hypothetical protein |
| 5G4_014 | 8276 | 9160 | PF03167.17,UDG |
| 5G4_015 | 9598 | 9362 | hypothetical protein |
| 5G4_016 | 9604 | 9792 | hypothetical protein |
| 5G4_017 | 12843 | 9862 | rRNA |
| 5G4_018 | 15224 | 13739 | rRNA |
| 5G4_019 | 15949 | 16818 | PF00941.19,FAD_binding_5;PF03450.15,CO_deh_flav_C |
| 5G4_020 | 16819 | 17301 | PF00111.25,Fer2;PF01799.18,Fer2_2 |
| 5G4_021 | 17414 | 19699 | PF01315.20,Ald_Xan_dh_C;PF02738.16,Ald_Xan_dh_C2 |
| 5G4_022 | 20028 | 19729 | hypothetical protein |
| 5G4_023 | 20383 | 20607 | PF01355.15,HIPIP |
| 5G4_024 | 20607 | 20729 | hypothetical protein |
| 5G4_025 | 20698 | 20895 | hypothetical protein |
| 5G4_026 | 20935 | 21588 | hypothetical protein |
| 5G4_027 | 21907 | 22710 | PF00990.19,GGDEF |
| 5G4_028 | 23846 | 22707 | PF01594.14,AI-2E_transport |
| 5G4_029 | 23957 | 24211 | hypothetical protein |
| 5G4_030 | 24264 | 25184 | PF04952.12,AstE_AspA |
| 5G4_031 | 25218 | 25667 | PF01475.17,FUR |
| 5G4_032 | 25704 | 26351 | PF13241.4,NAD_binding_7 |
| 5G4_033 | 26355 | 27266 | PF01379.18,Porphobil_deam;PF03900.13,Porphobil_deamC |
| 5G4_034 | 27263 | 28783 | PF00590.18,TP_methylase;PF02602.13,HEM4 |
| 5G4_035 | 28786 | 29259 | PF00293.26,NUDIX |
| 5G4_036 | 29447 | 29283 | hypothetical protein |
| 5G4_037 | 29596 | 30009 | PF13470.4,PIN_3 |
| 5G4_038 | 30026 | 30178 | PF00490.19,ALAD |
| 5G12_001 | 401 | 3 | PF00579.23,tRNA-synt_1b;PF01479.23,S4 |
| 5G12_002 | 1324 | 401 | hypothetical protein |
| 5G12_003 | 2086 | 1346 | hypothetical protein |
| 5G12_004 | 2109 | 2645 | PF01966.20,HD;PF13286.4,HD_assoc |
| 5G12_005 | 2657 | 2914 | hypothetical protein |
| 5G12_006 | 2924 | 3769 | PF03992.14,ABM |
| 5G12_007 | 3931 | 3816 | rRNA |
| 5G12_008 | 7733 | 3998 | rRNA |
| 5G12_009 | 9515 | 7981 | rRNA |
| 5G12_010 | 11000 | 9858 | hypothetical protein |
| 5G12_011 | 11303 | 13465 | PF03793.17,PASTA;PF03793.17,PASTA |
| 5G12_012 | 14021 | 13452 | PF13679.4,Methyltransf_32 |
| 5G12_013 | 15231 | 13990 | PF00763.21,THF_DHG_CYH;PF02882.17,THF_DHG_CYH_C |
| 5G12_014 | 16381 | 15254 | PF02245.14,Pur_DNA_glyco |
| 5G12_015 | 17304 | 16378 | PF00300.20,His_Phos_1 |
| 5G12_016 | 17824 | 17345 | PF01391.16,Collagen;PF01391.16,Collagen;PF01391.16,Collagen |
| 5G12_017 | 19365 | 17821 | hypothetical protein |
| 5G12_018 | 20501 | 19362 | PF07136.9,DUF1385 |
| 5G12_019 | 20504 | 21121 | hypothetical protein |
| 5G12_020 | 21656 | 21285 | hypothetical protein |
| 5G12_021 | 22207 | 21653 | hypothetical protein |
| 5G12_022 | 23664 | 22264 | PF00491.19,Arginase |
| 5G12_023 | 25094 | 23688 | PF13561.4,adh_short_C2 |
| 5G12_024 | 26497 | 25091 | PF05977.11,MFS_3 |
| 5G12_025 | 26813 | 26562 | PF01041.15,DegT_DnrJ_EryC1 |
| 5G12_026 | 27116 | 26913 | PF01435.16,Peptidase_M48 |
| 5G12_027 | 28034 | 27183 | PF04321.15,RmlD_sub_bind |
| 5G12_028 | 28035 | 29243 | PF04073.13,tRNA_edit |
| 5G12_029 | 29240 | 29542 | PF07992.12,Pyr_redox_2;PF02852.20,Pyr_redox_dim |
| 5G12_030 | 30315 | 29539 | PF00912.20,Transgly;PF00905.20,Transpeptidase;PF03793.17,PASTA |
| 5G12_031 | 30328 | 31284 | hypothetical protein |
| 5G12_032 | 32170 | 31286 | PF12760.5,Zn_Tnp_IS1595;PF12762.5,DDE_Tnp_IS1595 |
| 5G12_033 | 32982 | 32185 | PF00929.22,RNase_T;PF01541.22,GIY-YIG;PF02151.17,UVR |
| 5G12_034 | 33755 | 32979 | PF01680.15,SOR_SNZ |
| 5G12_035 | 34956 | 33961 | PF02934.13,GatB_N;PF02637.16,GatB_Yqey |
| 5G12_036 | 36632 | 34953 | hypothetical protein |
| 5G12_037 | 36859 | 36710 | hypothetical protein |
| 5G12_038 | 36867 | 37754 | hypothetical protein |
| 5G12_039 | 37960 | 37760 | hypothetical protein |
| 5G12_040 | 38159 | 38470 | hypothetical protein |
| 5G12_041 | 39538 | 38573 | PF01807.18,zf-CHC2 |
| 5G12_042 | 40116 | 39535 | PF00440.21,TetR_N;PF08359.9,TetR_C_4 |
| 5G12_043 | 40863 | 40177 | PF01425.19,Amidase |
| 5G12_044 | 42340 | 40955 | PF00313.20,CSD |
| 5G12_045 | 42549 | 43538 | PF02686.13,Glu-tRNAGln |
| 5H7_001 | 1 | 201 | hypothetical protein |
| 5H7_002 | 224 | 487 | hypothetical protein |
| 5H7_003 | 508 | 738 | hypothetical protein |
| 5H7_004 | 781 | 1194 | PF02657.13,SufE |
| 5H7_005 | 1694 | 1401 | PF03992.14,ABM |
| 5H7_006 | 2973 | 1702 | PF00067.20,p450 |
| 5H7_007 | 3321 | 2995 | PF00111.25,Fer2 |
| 5H7_008 | 3342 | 4124 | PF00440.21,TetR_N |
| 5H7_009 | 4915 | 4127 | PF00126.25,HTH_1;PF03466.18,LysR_substrate;PF03466.18,LysR_substrate |
| 5H7_010 | 5373 | 4954 | PF04379.12,DUF525 |
| 5H7_011 | 6661 | 5453 | PF01053.18,Cys_Met_Meta_PP |
| 5H7_012 | 6751 | 8034 | PF13231.4,PMT_2;PF16192.3,PMT_4TMC |
| 5H7_013 | 9154 | 8039 | PF00535.24,Glycos_transf_2;PF04138.12,GtrA |
| 5H7_014 | 10199 | 9156 | PF00180.18,Iso_dh |
| 5H7_015 | 10814 | 10215 | PF11967.6,RecO_N;PF02565.13,RecO_C |
| 5H7_016 | 11123 | 10866 | PF04380.11,BMFP |
| 5H7_017 | 11554 | 11441 | rRNA |
| 5H7_018 | 14464 | 11675 | rRNA |
| 5H7_019 | 16584 | 15110 | rRNA |
| 5H7_020 | 16985 | 16713 | hypothetical protein |
| 5H7_021 | 17285 | 18766 | PF13440.4,Polysacc_synt_3 |
| 5H7_022 | 18771 | 20717 | PF00884.21,Sulfatase |
| 5H7_023 | 20771 | 21679 | PF13650.4,Asp_protease_2;PF13650.4,Asp_protease_2 |
| 5H7_024 | 21745 | 23562 | PF00664.21,ABC_membrane;PF00005.25,ABC_tran |
| 5H7_025 | 23565 | 24410 | PF00781.22,DAGK_cat |
| 5H7_026 | 24971 | 24771 | PF08410.8,DUF1737 |
| 5H7_027 | 25354 | 24974 | PF05721.11,PhyH |
| 5H7_028 | 27571 | 26267 | PF00004.27,AAA;PF07724.12,AAA_2;PF10431.7,ClpB_D2-small |
| 5H7_029 | 28271 | 27708 | PF00227.24,Proteasome |
| 5H7_030 | 28870 | 28316 | PF04355.11,SmpA_OmlA |
| 5H7_031 | 28981 | 29496 | PF03981.10,Ubiq_cyt_C_chap |
| 5H7_032 | 29493 | 30020 | PF02620.15,DUF177 |
| 5H7_033 | 30589 | 30044 | PF00436.23,SSB |
| 5H7_034 | 32509 | 30668 | PF02421.16,FeoB_N;PF07670.12,Gate;PF07664.10,FeoB_C;PF07670.12,Gate |
| 5H7_035 | 32748 | 32506 | PF04023.12,FeoA |
| 5H7_036 | 33455 | 32799 | PF08511.9,COQ9 |
| 5H7_037 | 34097 | 33474 | PF13857.4,Ank_5 |
| 5H7_038 | 34162 | 34776 | PF02630.12,SCO1-SenC |
| 5H7_039 | 34764 | 35234 | PF03692.13,CxxCxxCC |
| 5H7_040 | 35221 | 35958 | PF01863.15,DUF45 |
| 5H7_041 | 36001 | 36432 | hypothetical protein |
| 5H7_042 | 36429 | 37418 | PF00383.21,dCMP_cyt_deam_1;PF01872.15,RibD_C |
| 5H7_043 | 37403 | 38020 | PF00677.15,Lum_binding;PF00677.15,Lum_binding |
| 5H7_044 | 38453 | 38052 | PF07238.12,PilZ |
| 5H7_045 | 39300 | 38587 | PF00155.19,Aminotran_1_2 |
| 5H7_046 | 39755 | 39339 | PF00155.19,Aminotran_1_2 |
| 5H7_047 | 39887 | 40330 | hypothetical protein |
| 5H7_048 | 40884 | 41600 | PF13610.4,DDE_Tnp_IS240 |
| 5H7_049 | 42008 | 42346 | PF01047.20,MarR |
| 5H7_050 | 42494 | 45274 | PF07715.13,Plug |
| 5H7_051 | 45351 | 46355 | hypothetical protein |
| 5H7_052 | 46394 | 48523 | hypothetical protein |
| 5H7_053 | 48659 | 50011 | hypothetical protein |
| 5H7_054 | 50409 | 50786 | PF00589.20,Phage_integrase |
| 5H7_055 | 50849 | 50983 | hypothetical protein |
| 5H7_056 | 51540 | 51058 | hypothetical protein |
| 5H7_057 | 51851 | 51654 | hypothetical protein |
| 5H7_058 | 52079 | 54166 | PF00933.19,Glyco_hydro_3;PF01915.20,Glyco_hydro_3_C;PF14310.4,Fn3-like |
| 5H7_059 | 54420 | 54175 | hypothetical protein |
| 5H7_060 | 54675 | 55424 | PF13683.4,rve_3 |
| 5H7_061 | 55421 | 55549 | PF13610.4,DDE_Tnp_IS240 |
| 5H7_062 | 55600 | 55842 | hypothetical protein |

a Blast results against pfam database by Hmmer
